# Double-strand break repair processes drive evolution of the mitochondrial genome in Arabidopsis

**DOI:** 10.1186/1741-7007-9-64

**Published:** 2011-09-27

**Authors:** Jaime I Davila, Maria P Arrieta-Montiel, Yashitola Wamboldt, Jun Cao, Joerg Hagmann, Vikas Shedge, Ying-Zhi Xu, Detlef Weigel, Sally A Mackenzie

**Affiliations:** 1 Center for Plant Science Innovation, University of Nebraska, N305 Beadle Center, Lincoln, NE 68588-0660, USA; 2 Max Planck Institute for Developmental Biology, Spemannstr. 35-39, 72076 Tübingen, Germany; 3 DuPont Agricultural Biotechnology, Experimental Station, Route 141 and Henry Clay Road, Wilmington, DE 19880-0357, USA

## Abstract

**Background:**

The mitochondrial genome of higher plants is unusually dynamic, with recombination and nonhomologous end-joining (NHEJ) activities producing variability in size and organization. Plant mitochondrial DNA also generally displays much lower nucleotide substitution rates than mammalian or yeast systems. Arabidopsis displays these features and expedites characterization of the mitochondrial recombination surveillance gene *MSH1 *(MutS 1 homolog), lending itself to detailed study of *de novo *mitochondrial genome activity. In the present study, we investigated the underlying basis for unusual plant features as they contribute to rapid mitochondrial genome evolution.

**Results:**

We obtained evidence of double-strand break (DSB) repair, including NHEJ, sequence deletions and mitochondrial asymmetric recombination activity in Arabidopsis wild-type and *msh1 *mutants on the basis of data generated by Illumina deep sequencing and confirmed by DNA gel blot analysis. On a larger scale, with mitochondrial comparisons across 72 Arabidopsis ecotypes, similar evidence of DSB repair activity differentiated ecotypes. Forty-seven repeat pairs were active in DNA exchange in the *msh1 *mutant. Recombination sites showed asymmetrical DNA exchange within lengths of 50- to 556-bp sharing sequence identity as low as 85%. *De novo *asymmetrical recombination involved heteroduplex formation, gene conversion and mismatch repair activities. Substoichiometric shifting by asymmetrical exchange created the appearance of rapid sequence gain and loss in association with particular repeat classes.

**Conclusions:**

Extensive mitochondrial genomic variation within a single plant species derives largely from DSB activity and its repair. Observed gene conversion and mismatch repair activity contribute to the low nucleotide substitution rates seen in these genomes. On a phenotypic level, these patterns of rearrangement likely contribute to the reproductive versatility of higher plants.

## Background

While producing a circular genetic map, plant mitochondrial chromosomes comprise a heterogeneous population of highly branched, circularly permuted linear molecules [1,2]. In plants, large-sized mitochondrial repeats (more than 1,000 bp) conduct high-frequency reciprocal recombination to subdivide the genome [3]. Given the linear structure of the genome, this recombination activity is suspected to participate in recombination-mediated replication initiation [4,5].

Mitochondrial genome plasticity is evident in extensive DNA polymorphism among closely related lines of a given plant species [6], rapid accumulation of polymorphisms within a line under cell culture conditions [7-9] and novel insertions, deletions and sequence chimeras [6]. These types of genomic alterations are causative in cytoplasmic male sterility (CMS) in a wide range of plant species [10], implying an adaptive rationale for the genomic phenomena [11]. Still, questions remain about the processes underlying mitochondrial genome variation, since most previous studies have addressed existing variations without the benefit of *de novo *events. Mitochondrial DNA replication in plants is not well-detailed, but both rolling circle and recombination-mediated replication have been suggested [4,12,13]. Likewise, the DNA repair mechanisms accounting for unusually low nucleotide substitution rates in plants are not known [14,15].

Sufficient mitochondrial DNA has been extracted from plant tissues to suggest the presence of multiple copies of the genome per cell, but available data suggest that the genome is organized into a heterogeneous population of DNA molecules maintained at vastly different relative copy numbers. Some substoichiometric DNA configurations are stably maintained in the genome and transmitted to progeny at levels approximating 1 copy per 200 cells [16]. The term "substoichiometric shifting" (SSS) refers to the reversible interconversion of these low-level forms to high copy numbers, influencing gene expression in the process [17,18]. This intragenomic copy number shifting is rapid, likely occurs during plant reproduction [19] and involves differential recombination at intermediate-sized repeats within the genome [20,21].

Asymmetrical recombination is characterized by the accumulation of only one of the two predicted DNA exchange products. Intermediate repeats support asymmetrical recombination in the plant mitochondrial genome at extremely low levels. This recombination activity accounts for faint "ghost" band polymorphisms commonly reported in DNA gel blot hybridization experiments involving long film exposure times [20]. Disruption of the Arabidopsis nuclear gene *MSH1 *(MutS 1 homolog), a component of mitochondrial recombination surveillance [20,22], allows increased recombination activity at these repeats. Recent studies of mitochondrial genomic changes in the Arabidopsis *msh1 *mutant, performed using PCR and DNA gel blot analysis, permitted functional identification of 33 intermediate repeat pairs 108 to 556 bp in size [21], implying a potential for massive rearrangement of the mitochondrial genome by this type of recombination activity. In "first-generation" *msh1 *mutants created by pollination of a wild-type line with mutant *msh1 *pollen and selection of the F_2 _*msh1/msh1 *segregant, low-level recombination activity is initiated at each repeat. By the "advanced generation," derived by recurrent self-pollination of the *msh1 *mutant, recombination is extensive and recombinants are present at high copy numbers [21].

Most previously acquired information about plant mitochondrial genome variation has relied on studies of CMS mutants and DNA polymorphism in natural populations. Discovery of *msh1 *permits direct investigation of mitochondrial *de novo *recombination and its relationship to high levels of structural variation and unusually low nucleotide substitution rates unique to plants. In our present study, next-generation sequencing provided sufficient sensitivity for detailed investigation of the asymmetrical strand invasion and gene conversion process as well as sufficient throughputfor cataloging genomic alterations associated with rapid, intraspecific mitochondrial genome evolution.

## Results

### Next-generation sequence analysis reveals additional intermediate repeats not previously detected

**Figure 1:**
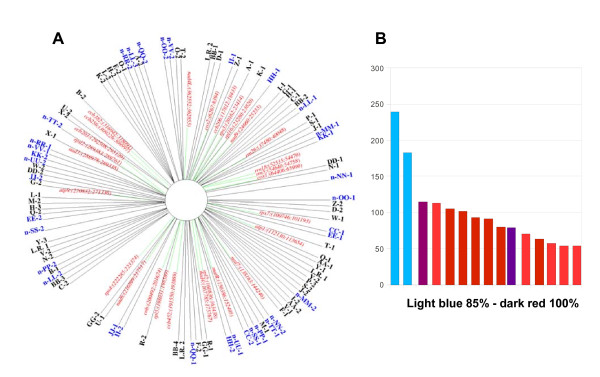
**Identification of 14 additional Arabidopsis mitochondrial genome repeat pairs**. **(A) **Map of intermediate mitochondrial repeats that are active in the *msh1 *mutant. Newly identified repeats are shown in blue. **(B) **Size (*y*-axis) and percentage homology (color) distribution of newly identified repeats.

Mitochondrial asymmetric DNA exchange in the *msh1 *mutant involves 47 repeat pairs ranging from 50 to 556 bp in size (Figure 1A). This was deduced by identification of paired adjacent sequences that were known to map distal to one another. Such paired-end sequences were clustered in *msh1 *first- and advanced-generation mutants in a manner distinct from the wild type (Additional file 1, Figure S1). All identified clusters could be explained by recombination events involving intermediate repeats. While confirming previously identified repeats [21], deep sequencing revealed 14 new repeat pairs ranging from 50 to 250 bp in size, with sequence homology within each pair ranging from 85% to 98% and the smallest repeats displaying the highest identity (Figure 1B). All recombination detected at intermediate repeats showed asymmetrical exchange activity.

### Intermediate repeats reveal essential features of the mitochondrial DNA exchange process

**Figure 2:**
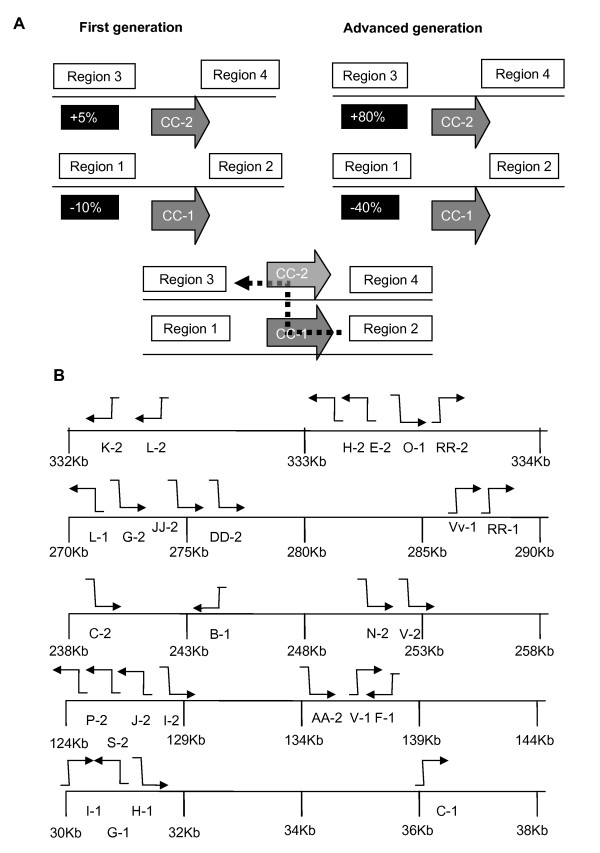
**Asymmetrical strand invasion polarity at intermediate mitochondrial repeats**. **(A) **Relative changes from wild type in deep sequence coverage across each environment for repeat CC in *msh1 *first-generation (left) and advanced-generation (right) mutants were used to define strand invasion polarity. Polarity shown in the lower panel is based on depletion of region 1 relative to region 2 compared to wild type and on an increase in region 3 relative to region 4 compared to wild type. To quantify read depth of flanking regions, we considered all read pairs for which one end mapped to the repeat and the other mapped to the region flanking the repeat. Thus, the length of the flanking sequence is approximately 220 bp. **(B) **Graphical representation of strand invasion polarities across clusters of repeats in the Arabidopsis Col-0 mitochondrial genome. Polarities were defined by deep sequence analysis using the procedure shown in **(A) **and confirmed by DNA gel blot analysis. Polarity of strand invasion was established on the basis of which repeat-flanking region displayed an increase in sequence depth and, in the case of class I repeats, a concomitant decrease in sequence depth across the other repeat-flanking region.

The size range of active repeats reflects the length of homology required to permit DNA exchange. While repeated sequences smaller than 50 bp were detected in the genome, they showed no evidence of recombination activity, suggesting that 50 bp approximates a lower size limit for *msh1-*associated DNA exchange. Recombination patterns at intermediate repeats imply a process of break-induced replication on the basis of the uniformly asymmetrical nature of DNA exchange [23,24]. Each repeat showed a conserved polarity for strand invasion, which was suggested by the accumulation of only one of the recombinant products and, in some cases, concomitant loss of one of the parental forms [21]. The observed polarity was confirmed by comparing high-throughput sequence frequencies at sites flanking repeats in first- and advanced-generation *msh1 *mutants with the wild type (Figure 2A; Additional file 2, Figure S2; and Additional file 3, Table S1). Analysis of clustered intermediate repeats allowed us to compare relative strand invasion polarities and showed both opposite and same-strand polarities at neighboring repeats, with no obvious association with local direction of transcription (Figure 2B). It is possible that these sites of active, bidirectional strand invasion might represent replication origins (for example, at sites between repeats E-2 and O-2 and between repeats L-1 and G-2).

**Figure 3:**
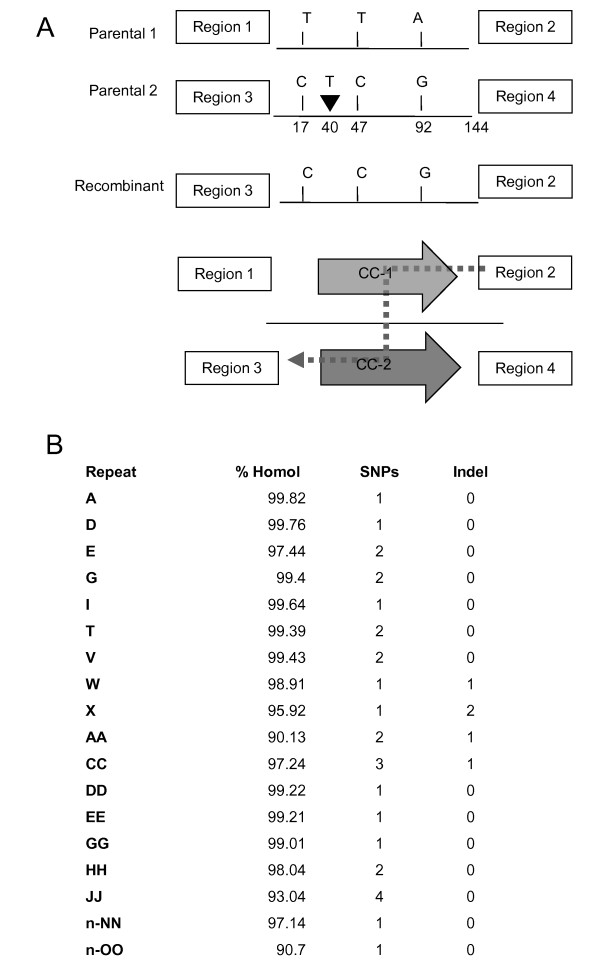
**Evidence of mismatch repair associated with DNA exchange at intermediate repeats in the *msh1 *mutant**. **(A) **Four polymorphisms differentiate repeat CC-1 from CC-2, three SNPs and one single-nucleotide insertion. The recombinant sequence suggests a process of mismatch repair following heteroduplex formation. **(B) **List of nonidentical repeats where gene conversion was observed in the recombinant product. Homology between copies of each repeat, as well as the number of corrected SNPs and indels, are indicated. In the case of each repeat listed in the table, mismatches were resolved in favor of the putative donor strand, which were calculated using data from the frequencies of SNPs for each of the repeats in the different generations of the *msh1 *mutant. No clear pattern (donor vs recipient strand) for indel resolution was observed.

Sequence analysis of the recombination product for nonidentical repeats, composing about 50% of all active intermediate repeats, provided evidence of heteroduplex formation followed by mismatch repair (Figures 3A and 3B). According to our data, nucleotide mismatches are repaired using the donor strand as a template. In Figure 3A, the nucleotide insertions in the donor strand are cleaved, giving the appearance of partial gene conversion. However, a more extensive survey of repeats showed no clear pattern of donor or recipient strand cleavage for correction of nucleotide insertions. These results reveal the presence of a mitochondrial mismatch repair mechanism in plants that is independent of *MSH1*.

**Figure 4:**
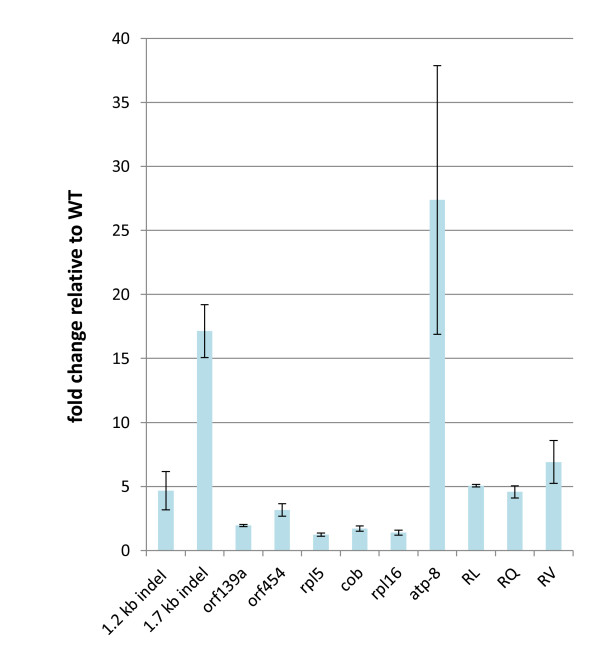
**Graphical presentation of results from quantitative PCR to assess changes in relative copy number of 11 different regions of the mitochondrial genome in wild-type (Col-0) and *msh1 *mutant lines**. Error bars show standard errors for three biological replicates with quantitative PCR results from Col-0 set at 1. "HCN" indicates high copy number version of *Atp8*.

To assess the influence of *msh1 *recombination on mitochondrial genome copy number, we compared relative copy number at 11 sites within the mitochondrial genome of the wild type (Col-0) and the *msh1 *mutant (Figure 4). In each case, copy number was higher in the mutant, implying that overall mitochondrial DNA copy number increases with enhanced recombination. One possible explanation for this observation might be enhanced break-induced replication of the genome in the absence of MSH1

### Mitochondrial recombination in the *msh1 *mutant produces two patterns of genomic rearrangement

**Figure 5:**
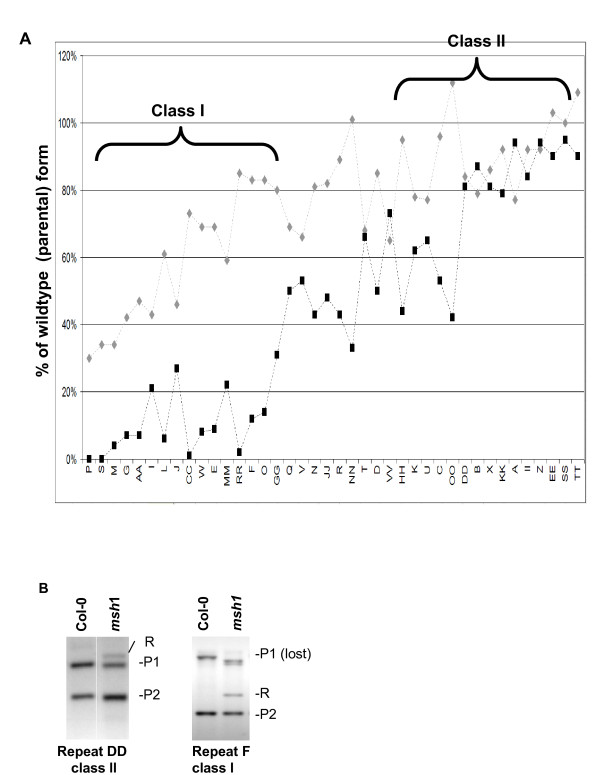
**Deducing the fate of the parental form following recombination**. **(A) **Graph depicting deep sequence coverage of the least predominant environment (the invading strand parental form) around each repeat. This is measured by quantifying the changes in read depth across flanking regions compared to wild type. Repeats were sorted by average activity, with first-generation *msh1 *repeats shown in gray and advanced-generation results shown in black. Class I repeats show significant reduction of the invading strand parental form in the resulting recombinant, and class II repeats retain the parental form in high copy numbers following recombination. Mapping of class I repeats gives the appearance of deletion in the recombinant. **(B) **DNA gel blot hybridization experiments with repeat DD and repeat F as probes show examples of class I and class II recombination outcomes in an advanced-generation *msh1 *mutant. P1 and P2 designate parental configurations, and R is the recombinant form. Additional bands in the *msh1 *lane probed with repeat F represent secondary recombinations, defined as events that depend on novel genomic environments created by primary recombination to occur.

We compared the read frequency for parental forms in different generations of the *msh1 *mutant against wild type. This allowed us to trace the fate of parental products over time in the *msh1 *mutant. Most repeats appeared to fall into one of two general categories (Figure 5). Class I repeats lost one parental form as they acquired the recombinant, resulting in the appearance of both deletion and gain of genomic forms. Class II repeats retained the parental forms as they acquired the recombinant, resulting in the appearance of a novel genomic form. The parental form that was reduced was consistently that which donates the invading strand.

This "gain" and "loss" of genetic environments is the consequence of SSS, and PCR-based analysis has revealed that these genomic environments change in relative copy number merely as a consequence of recombination [20,21]. From a genomic perspective, sequences residing in proximity to these intermediate repeats have the greatest likelihood of undergoing SSS, although we have identified sequences some distance from an identifiable repeat that also undergo SSS. We observed several essential genes residing in proximity to class II repeats (Table 1), but none residing adjacent to class I repeats (with the exception of *Atp9*, which encompasses a class I repeat). Presumably, the relative proximity of a repeat to essential genetic information could impose selection for the parental form, the class II pattern.

**Table 1:** Proximity of class II repeats to known genes

Repeat	Position	Gene	Gene position
B	322,365	*Cox3*	330,907
X	289,252	*Nad3*	288,978
KK	52,356	*Rrn26*	40,047
A	19,679	*Rps3*	23,280
II	210,316	*Nad6*	216,899
Z	11,399	*Ccb206*	17,812
EE	107,016	*Rps7*	101,192
SS	263,498	*Atp6-1*	253,200

In *msh1 *advanced- versus first-generation mutants, we investigated the reversibility of recombination activity upon replacement of the *MSH1*allele. While first-generation *msh1 *rearrangements appeared to be fully reversible by crossing the *msh1 *line with the wild type and deriving an F_2 _progeny, advanced-generation rearrangements were not reversible, based on analysis of 10 individual F_2 _plants from both crosses and assaying three repeats (L, D and F) for recombinant configurations (data not shown). We attribute these results to cytoplasmic sorting of the least prevalent forms once *MSH1 *is reintroduced. Thus, in the first generation, where the quantity of recombinant molecule is low relative to nonrecombinant, recombinant forms are diluted and nonrecombinant forms predominate. In advanced generations, where recombinant forms have accumulated to higher levels, their loss by sorting is precluded.

### The Arabidopsis mitochondrial genome undergoes rapid and extensive rearrangement

**Figure 6:**
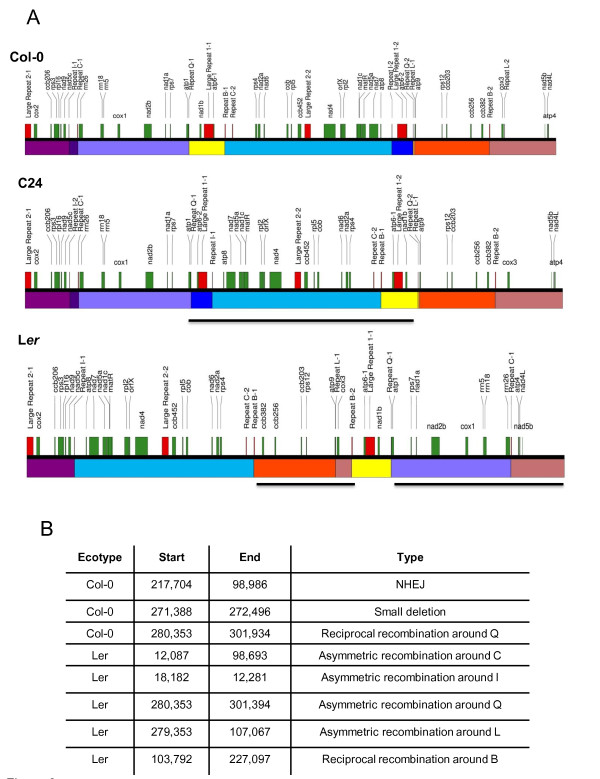
**Mitochondrial genome rearrangements in ecotypes Col-0, C24 and L*er***. **(A) **Genome maps are constructed from sequence data. Regions are color-coded, with genes shown in green and selected repeats shown in red. Underlying bars represent regions that are inverted in orientation relative to the genomic map of Col-0. **(B) **List of structural variations in Col-0 and Landsberg *erecta *(L*er*) genes relative to C24. The position and type of polymorphism are shown.

In our investigation of the influence of double-strand break (DSB)-mediated changes on genomic variation, we sequenced and assembled the mitochondrial genomes of ecotypes Col-0 and Landsberg *erecta *(L*er*) and compared these with the publicly available C24 sequence (confirmed by resequencing). Among the three ecotypes, we found virtually identical genomic coding capacity within genomes that had undergone extensive rearrangements. The majority of the structural polymorphisms found can be attributed to DSB repair activity by either nonhomologous end-joining (NHEJ) or intermediate-sized repeat-mediated recombination (Figures 6A and 6B).

Stoichiometric variation among ecotypes includes a repeat-containing interval of 1.7 kb, originally reported by Forner *et al*. [25], that is present in Col-0 but substoichiometric in C24 and L*er *[21]. The corresponding "junction" configuration that lacks this 1.7-kb insertion is present in C24 and L*er*, but is substoichiometric in Col-0. Likewise, an unrelated interval of 1.2 kb present in C24 and L*er *was found to be substoichiometric in Col-0. The corresponding "junction" lacking this 1.2-kb interval is present in Col-0 but substoichiometric in C24 and L*er *(data not shown).

While cross-ecotype genomic comparisons gave evidence of ecotype-specific divergence by insertion and deletion of distinct genetic information, this variation was largely accounted for by asymmetrical recombination-mediated SSS. Researchers in earlier cross-genotype studies in maize and sugar beet, involving multiple lines, reported similar mitochondrial variation [6,26]. While the researchers in neither of the earlier studies investigated substoichiometric forms in the genome, we suggest that some of the reported insertions and deletions distinguishing the closely related genotypes in those species likely derive from SSS activity.

The extensive mitochondrial DNA polymorphism among lines within a species was not surprising, given what has been reported in other plant studies [27]. What was significant in our investigation was the discovery that nearly all of the variation observed could be accounted for by DSB repair activity or nucleotide substitutions. This observation prompted us to investigate the extent to which DSB repair processes have participated in plant mitochondrial genome evolution.

### DSB repair occurs widely in Arabidopsis mitochondrial genomes

**Figure 7:**
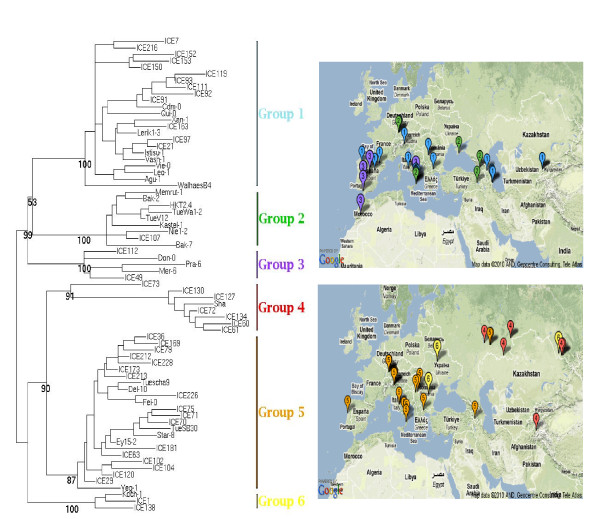
**Phylogenetic analysis of 72 ecotypes based on mitochondrial SNPs suggests division into six groups in the left panel**. The tree shown is unrooted. The right panel shows the geographic distribution of the depicted groups.

To assess the role of DSB repair in mitochondrial genome evolution, we assembled and compared mitochondrial sequences from 72 different Arabidopsis ecotypes. We collaborated with the Arabidopsis 1001 Genomes project (http://www.1001genomes.org/) to obtain mitochondrial sequences for subsequent identification of polymorphisms. The mitochondrial genome sequences were scanned for SNPs to assess phylogenetic relationships among the ecotypes. Cross-ecotype SNP comparisons suggested that the 72 ecotypes comprised six phylogenetic groups (Figure 7). The derived groupings appeared to correspond generally with the geographic origin of each line, and the variation measured is likely to reflect divergence over a span of 120,000 years [28]. This observation would be consistent with the observed sequence divergence across ecotypes, which was approximately one-twentieth that of *Arabidopsis lyrata*, with an estimated divergence time of four to five million years.

**Figure 8:**
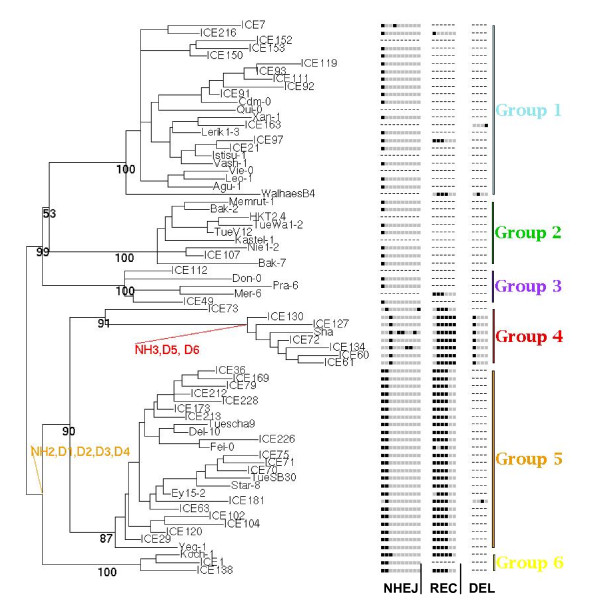
**Mitochondrial genome structural changes coincide with SNP-based phylogeny**. Structural changes arising by nonhomologous end-joining (NHEJ), deletions caused by recombination at repeats (REC) and small deletions, perhaps arising by replication slippage (DEL), were categorized according to ecotypes, allowing assessment of the relationship of SNP-based phylogeny to changes arising by genomic rearrangement. Putative sites in the tree where some of the changes may have occurred are indicated. The tree shown is unrooted.

Mitochondrial structural polymorphisms revealed extensive genomic variation distinguishing each line. The majority of structural changes represented nonhomologous, recombination or small deletion activity (Figure 8). The small deletions were a small proportion (about 20%) of the total structural polymorphisms detected. Cross-ecotype cataloging of observed structural variation revealed remarkable correspondence with the six SNP-based phylogenetic groups (Figure 8). These observations would be expected if DSB repair served as a prominent contributor to the rapid evolution rate of plant mitochondrial genomes.

## Discussion

While we have not yet identified the genomic features that condition asymmetrical recombination in the Arabidopsis mitochondrial genome, we have shown in the present study that the process comprises the primary pattern of DNA exchange at intermediate-sized repeat sequences in the absence of *MSH1*. It is possible that the observed asymmetrical exchange might be a consequence of differential maintenance of recombination products rather than the consequence of dominant strand invasion polarity. While it has been suggested that analysis of low-frequency recombination events can sometimes be confused with PCR-generated artefacts [29], our analysis of *de novo *recombination in *msh1 *confirms prolific DNA exchange activity. Data from our studies suggest that strand invasion occurs throughout the genome, a likely consequence of random DSBs, but is prevented by *MSH1 *from producing successful DNA exchange in stretches of sequence homology under about 556 bp. In the absence of *MSH1*, strand invasion proceeds at smaller lengths of homology to a lower limit of approximately 50 bp.

In the wild-type line, mitochondrial DNA exchange at intermediate repeats does occur, but at very low levels, presumably because it is prevented by MSH1 surveillance. This infrequent recombination is detected as faint ghost bands by DNA gel blot analysis [20,21]. *MSH1 *encodes a carboxy-terminal GIY-YIG endonuclease domain, although direct participation in double-strand cleavage to abort DNA exchange has not yet been formally tested. MSH1 presumably permits repair of a DSB at stretches of homology greater than 550 bp following heteroduplex formation, assuming a break-induced replication model (see Additional file 4, Figure S3).

In the *msh1 *mutant, heteroduplex formation occurs within the intermediate repeat stretches of homology. The observation of heteroduplex-mediated mismatch repair in this study provides important evidence relevant to the low mitochondrial nucleotide substitution rates reported in plants [14]. Our study also shows that this mismatch repair activity is independent of *MSH1*. Interestingly, nucleotide divergence within intermediate repeat intervals, estimated across 72 Arabidopsis ecotypes at 5.5 per 10,000 bp, approximates the average for the genome as a whole, which is 5 per 10,000 bp. However, in regions of the genome where high-frequency reciprocal DNA exchange occurs, within large (more than 1,000 bp) repeats, levels of polymorphism were 66 times lower, averaging 7.5 per Mbp for the 6.5-kb repeat across 72 ecotypes. These observations imply that the low-frequency exchanges observed at intermediate repeats in the wild-type line may be sufficient to confer the low nucleotide substitution rate observed generally in the Arabidopsis mitochondrial genome. In animal lineages, nucleotide substitution rates in the mitochondrial genome are much higher, with the notable exception of corals and their relatives [30]. Intriguingly, the corals appear to be the only known animal lineage encoding a mitochondrial Msh1, with similarities in structure to MSH1 in plants [31].

**Figure 9:**
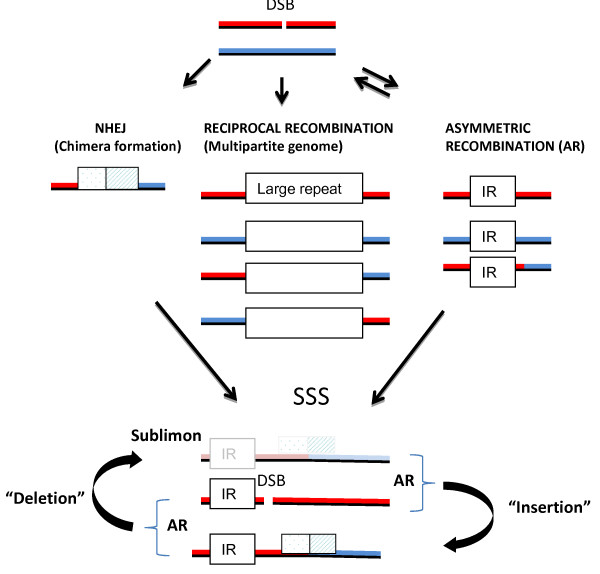
**Modeling alternative double-strand break repair outcomes**. Sequence analysis suggests three means of double-strand break (DSB) repair, resulting in evidence of nonhomologous end-joining (NHEJ), reciprocal recombination at large repeats and asymmetrical recombination at intermediate repeats (IRs). Asymmetrical recombination activity at IRs residing on molecules containing gene chimeras can result in substoichiometric shifting (SSS) for relative copy number suppression or amplification of the genomic environment surrounding the chimera.

Analysis of mitochondrial genomic rearrangements differentiating the Col-0, L*er *and C24 ecotypes revealed three primary classes of genomic changes as recombination, NHEJ and deletion, all of which may represent consequences of DSB repair (Figure 9). An important aspect of NHEJ may be the formation of sequence chimeras, some of which encode transcriptionally active open reading frames that could condition phenotypic variations, such as CMS. Proximity of a CMS sequence chimera to a class I repeat can allow changes in copy number of the region by recombination at the repeat. CMS induction was shown to occur by *MSH1 *suppression in various plant species [32]. The resulting mitochondrial DNA rearrangements are presumed to result in copy number amplification of a novel, preexistent, CMS-inducing genomic environment.

SSS-associated copy number reduction of male sterility sequences has been associated with spontaneous reversion to fertility in common bean, *Brassica*, and maize S-type cytoplasm [18,33-35]. Whether the frequency of reversion responds to altered *MSH1 *expression in the plant, and whether *MSH1 *expression is influenced by environmental cues such as unsuccessful pollination, is an intriguing possibility. *MSH1 *transcription is environmentally responsive [36], but it is not clear whether carbon flux acts as a signal. An isolated male-sterile plant, following a period of unsuccessful pollination, might affect carbon signaling to suppress *MSH1*, amplifying mitochondrial recombination for SSS and reversion to fertility. Such a process, if it occurred late in the flowering cycle, would ensure a successful seed set.

## Conclusions

In our present study, we conducted a detailed analysis of *de novo *recombination and genome rearrangement processes that characterize plant mitochondrial genomes. The rapid rate of plant mitochondrial intraspecific structural variation and low nucleotide substitution rates can both be accounted for by unusual DSB repair within the genome. These observations consequently implicate DSB repair processes as a driver in the evolutionary divergence of plant and animal mitochondria. The dynamic nature of plant mitochondrial genomes, and their ability to retain genetic variation substoichiometrically, presumably enhance environmental responsiveness and increase reproductive versatility.

## Methods

### DNA gel blot and PCR assays

Total genomic DNA was extracted from above-ground tissues according to previously published procedures [37]. Mitochondrial DNA preparation, DNA gel blot analysis and hybridizations were performed as described previously [38]. The primers used to PCR-amplify probes are listed in Additional file 5, Table S2.

### Quantitative polymerase chain reaction

Genomic DNA (100 ng) was used for quantitative PCR with the SYBR GreenER qPCR SuperMix for iCycler kit (Invitrogen, Carlsbad, CA, USA). Quantitative PCR data were collected and analyzed using iCycler iQ version 3.1 software (Bio-Rad Laboratories, Hercules, CA, USA). Three biological replicates were used for each experiment, each sample was run in triplicate and the results were averaged. Nuclear single-copy loci (At*MSH1 *and At*ACTIN2*) served as nuclear DNA content controls. The primers used for real-time analysis are presented in Additional file 5, Table S2.

### Sequencing and read mapping

Illumina Solexa Sequencing Technology (Illumina, Inc, San Diego, CA, USA) paired-end reads of Col-0 were assembled using the comparative assembler AMOScmp version 2.08 software program (with default parameters) [39] with the C24 mitochondrial genomic sequence as a baseline to obtain 57 contigs with an N50 of 25 kb and coverage of 230×. In a similar manner, C24 was resequenced and 54 contigs obtained, with an N50 of 30 kb and coverage of 371×. Single and paired-end reads for L*er *were mapped against the reference genome of C24 using Velvet version 1.1 software (with default parameters) [40] and SHOREmap version 0.5 software (with default parameters) [41] to obtain 19× coverage, and consensus sequences and deleted regions were identified using custom-made Perl scripts. The contigs for these three ecotypes were assembled into circular scaffolds based on DNA gel blot analysis and PCR assays. Graphical genome maps were generated using OGDraw software [42]. The annotated genome sequences for ecotypes C24, L*er *and Col-0 are available from GenBank [GenBank:JF729200, GenBank:JF729201, GenBank:JF729202], and intermediate repeats are annotated. Generated raw sequence data are available from the sequence read archive [GenBank:SRA039856].

The Illumina Genome Analyzer (Illumina, Inc) was used with paired-end reads of 36 bp, a mean library length of 220 and a standard deviation of about 20. This length spanned approximately half of the repeats in the mitochondrial genome. Coverage for first-generation *msh1 *was about 600×{and for advanced-generation *msh1 *it was about 200×. Reads of the Illumina Solexa Sequencing Technology paired-end sequencing for first- and advanced-generation *msh1 *(Col-0) mutants were mapped to the mitochondrial genome of Col-0 using Velvet software [40] and SHOREmap software [41], and recombination points were identified with a customized script designed to cluster paired-end reads whose ends map to regions separated by more than 1,000 bp. Clusters with more than 10 reads in both the first- and advanced-generation *msh1 *lines were selected, and putative repeats were identified by BLAST analysis [43] of the selected region against the mitochondrial map. Coverage analysis of the regions around the intermediate repeats was done using custom-made scripts. On the basis of observed behavior in both the first- and advanced-generation *msh1 *lines and DNA gel blot data, we determined the putative direction of strand invasion around the repeats. For nucleotide sequence analysis of the intermediate repeats in the *msh1 *mutants, we used a custom-made script to derive the consensus sequence.

Illumina Solexa Sequencing Technology paired-end sequence data for 80 ecotypes was produced by the Weigel laboratory at the Max Planck Institute for Developmental Biology (http://www.1001genomes.org/). Aligned sequence data against the C24 mitochondrial genome with SHOREmap version 0.5 software [41] allowed us to select of 72 ecotypes with more than 15× coverage. SNPs against C24 were calculated using custom-made scripts for regions with more than 5× coverage and nucleotide occurrence in more than 80% of the reads mapped. For phylogenetic analysis, a concatenated set of 1,059 SNPs distributed across the genome in both protein-coding regions and intergenic regions was considered for each ecotype. Poorly aligned regions and regions with gaps in most ecotypes were excluded from the analysis, and a total of 676 nucleotides were used for each ecoytpe. A maximum likelihood model was preferred over a parsimony approach, considering its statistical consistency. Using PhyML version 3.0 software [44], we constructed the tree with an HKY85 substitution model and a γ distribution with four different categories, and one hundred bootstrap replicates were performed. The resulting trees are unrooted. Graphical phylogenetic trees were generated by using TreeDyn software [45]. Geographical maps were generated by using a custom-made script and Google Static Maps API version 2 (http://code.google.com/apis/maps/documentation/staticmaps/). Deletions were identified by analyzing the coverage data, and recombination polymorphisms were identified by grouping paired-end reads whose ends map to regions separated by more than 1,000 bp with a custom-made script. Data for the 80 ecotypes are available at "1001 Genomes: A Catalog of *Arabidopsis thaliana *Genetic Variation: MPICao2010: 80 *Arabidopsis thaliana *accessions" (http://www.1001genomes.org/projects/MPICao2010/index.html) [46].

## Abbreviations

bp: base pair; kb: kilobase; Mbp: megabase pair; PCR: polymerase chain reaction; SNP: single-nucleotide polymorphism.

## Conflicts of interests

The authors declare that they have no competing interests.

## Authors' contributions

JID participated in computational analysis, genome assembly, literature screening and manuscript preparation. MAM carried out PCR and gel blot hybridization analyses and genome mapping. YW, VS and YZX participated in mitochondrial genome sequencing experiments in *msh1*, Col-0, L*er*, and C24. JC, JH and DW generated total genomic sequence from 72 Arabidopsis ecotypes. SAM designed and coordinated the study, participated in data analysis and prepared the manuscript. All authors read and approved the final manuscript.

## Supplementary Material

Additional file 1 **Figure S1. Clusters of recombinant paired ends in wild-type and *msh1 *first- and advanced-generation mutants reveal recombination differences**. *x*- and *y*- axes represent the coordinates of the first part and second part, respectively, of the paired-end read. The radius of the circle represents the number of reads that cluster around such coordinates. The graphs correspond to wild-type **(a)**, *msh1 *first-generation **(b) **and *msh1 *advanced-generation lines **(c)**. Small-radius circles in the wild-type lines were taken as background and subtracted from the graphs of the mutants. Increased levels of recombination are observed across generations of the mutant. The red circle represents a structural feature present in wild types and mutants at the same level used as controls. Click here for file

Additional file 2 **Figure S2. Fate of genomic environments around repeats in *msh1 *mutants across generations**. *y*-axis represents the percentage changes in coverage across each copy of the repeat with respect to wild type. The pink and red squares represent the recombinant forms gained in *msh1 *first- and advanced-generation lines. The turquoise and blue triangles represent the parental forms lost in *msh1 *first and advanced generations. These were calculated as the difference in coverage of right versus left flanking regions of each repeat relative to wild type. A gradient, as well as a correspondence, between gain of recombinant and loss of parental forms is observed across generations. Click here for file

Additional file 3 **Table S1. Changes in coverage and putative strand invasion polarity for intermediate repeats in the *msh1 *mutant**. Coverage data are shown for first- and advanced-generation mutants. The data show changes in coverage with respect to Col-0 for regions flanking each repeat, along with deduced order of strand invasion (strand polarity). Confirmation by gel blot analysis is indicated for several sites. Click here for file

Additional file 4 **Figure S3. Potential outcomes of double-strand break repair during break-induced replication**. With the occurrence of a double-strand break (DSB) during replication, 5' to 3' strand recession occurs, followed by strand invasion to a homologous duplex for reestablishment of the replication fork. **(1) **Occurrences of the DSBs at a unique region. **(2) **When the DSB occurs at a repeated region, two outcomes are possible: **(a) **strand invasion at homologous repeats or **(b) **strand invasion across different copies of the repeat to produce a new genomic environment as the recombinant *MSH1 *prevents DNA exchange beyond the region of sequence homology within the repeated sequence. Click here for file

Additional file 5 **Table S2. Primers used in the study**. Click here for file
