# Supplementary figures and images for: Double-strand break repair processes drive evolution of the mitochondrial genome in Arabidopsis
Source: BMC Biol. 2011 Sep 27;9:64. doi: 10.1186/1741-7007-9-64 (PMC3193812; doi:10.1186/1741-7007-9-64)

Figure S1

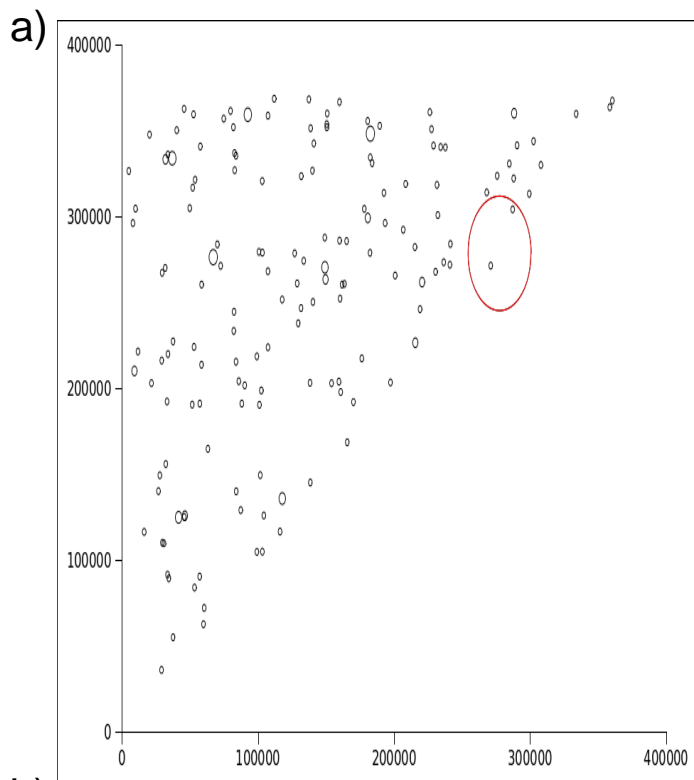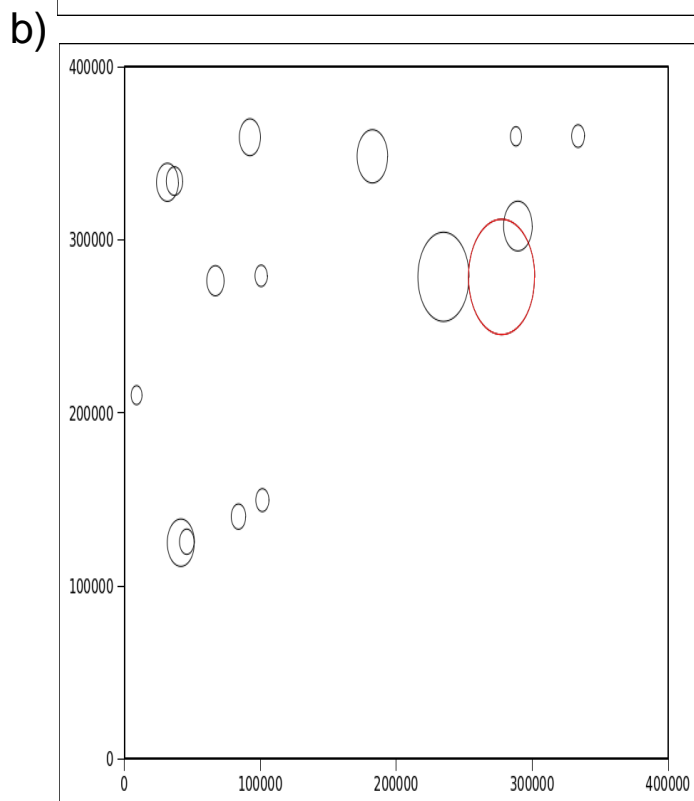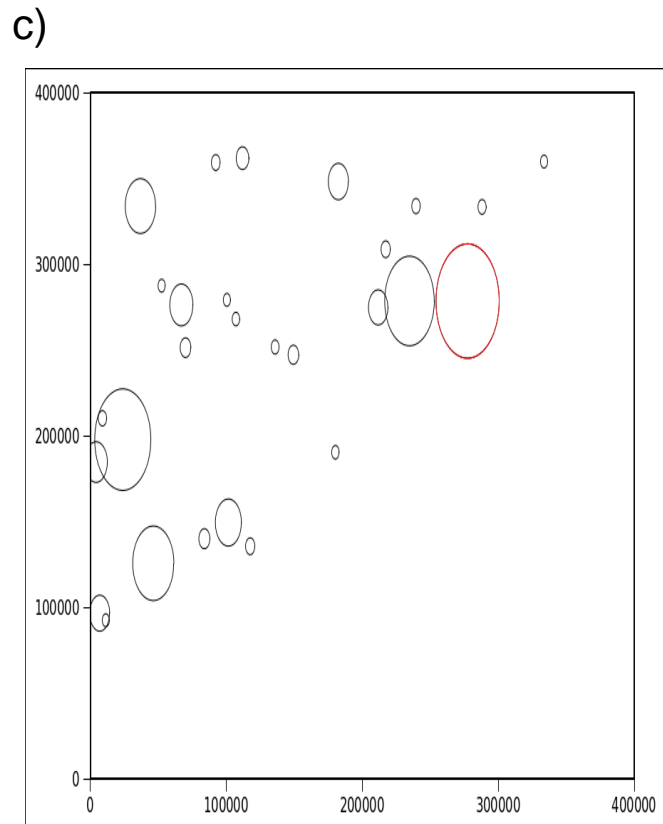

Supplement: Additional file 1 — Figure S1. Clusters of recombinant paired ends in wild-type and msh1 first- and advanced-generation mutants reveal recombination differences. x- and y- axes represent the coordinates of the first part and second part, respectively, of the paired-end read. The radius of the circle represents the number of reads that cluster around such coordinates. The graphs correspond to wild-type (a), msh1 first-generation (b) and msh1 advanced-generation lines (c). Small-radius circles in the wild-type lines were taken as background and subtracted from the graphs of the mutants. Increased levels of recombination are observed across generations of the mutant. The red circle represents a structural feature present in wild types and mutants at the same level used as controls. [file 1741-7007-9-64-S1.PDF]

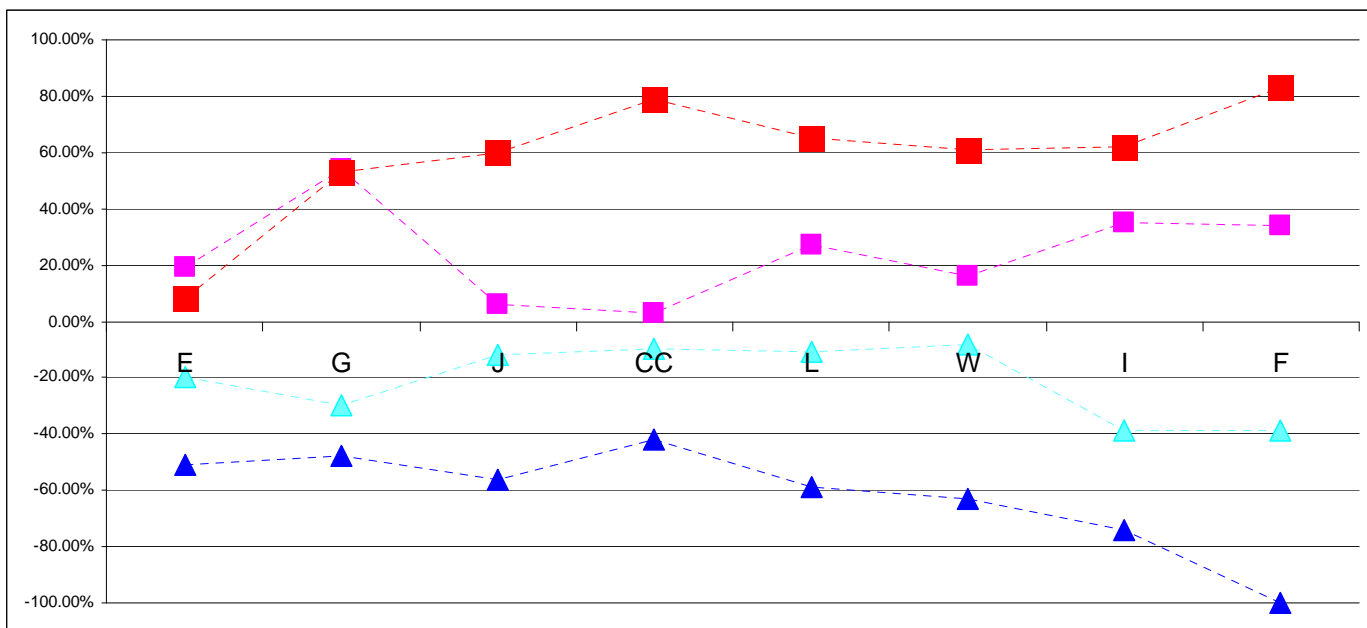

Figure S2

Supplement: Additional file 2 — Figure S2. Fate of genomic environments around repeats in msh1 mutants across generations. y-axis represents the percentage changes in coverage across each copy of the repeat with respect to wild type. The pink and red squares represent the recombinant forms gained in msh1 first- and advanced-generation lines. The turquoise and blue triangles represent the parental forms lost in msh1 first and advanced generations. These were calculated as the difference in coverage of right versus left flanking regions of each repeat relative to wild type. A gradient, as well as a correspondence, between gain of recombinant and loss of parental forms is observed across generations. [file 1741-7007-9-64-S2.PDF]

## Slide 1
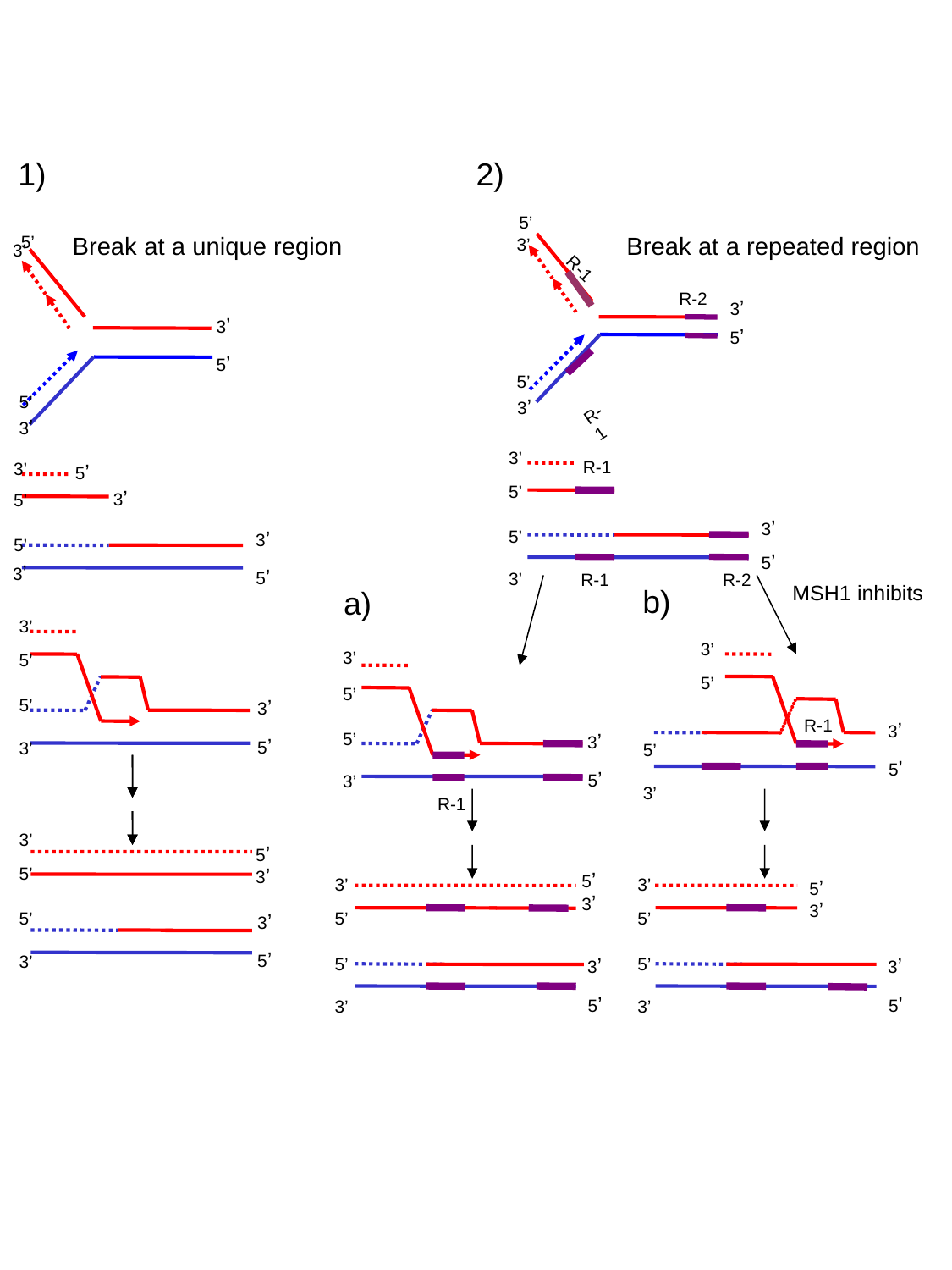

1)
2)
5’
5’
Break at a unique region
3’
Break at a repeated region
3’
R-1
R-2
3’
3’
5’
5’
5’
 R-1
5’
3’
3’
3’
3’
5’
R-1
5’
3’
5’
3’
3’
5’
5’
5’
3’
5’
3’
R-1
R-2
b)
MSH1 inhibits
 a)
3’
3’
3’
5’
5’
5’
5’
3’
R-1
3’
5’
3’
5’
3’
5’
5’
5’
3’
3’
R-1
3’
5’
5’
3’
5’
3’
3’
5’
3’
3’
5’
3’
5’
5’
5’
3’
5’
3’
5’
3’
5’
5’
3’
3’

Supplement: Additional file 4 — Figure S3. Potential outcomes of double-strand break repair during break-induced replication. With the occurrence of a double-strand break (DSB) during replication, 5' to 3' strand recession occurs, followed by strand invasion to a homologous duplex for reestablishment of the replication fork. (1) Occurrences of the DSBs at a unique region. (2) When the DSB occurs at a repeated region, two outcomes are possible: (a) strand invasion at homologous repeats or (b) strand invasion across different copies of the repeat to produce a new genomic environment as the recombinant MSH1 prevents DNA exchange beyond the region of sequence homology within the repeated sequence. [file 1741-7007-9-64-S4.PPT]
