# Supplementary material for: Double-strand break repair processes drive evolution of the mitochondrial genome in Arabidopsis
Source: BMC Biol. 2011 Sep 27;9:64. doi: 10.1186/1741-7007-9-64 (PMC3193812; doi:10.1186/1741-7007-9-64)
Supplement: Additional file 3 — Table S1. Changes in coverage and putative strand invasion polarity for intermediate repeats in the msh1 mutant. Coverage data are shown for first- and advanced-generation mutants. The data show changes in coverage with respect to Col-0 for regions flanking each repeat, along with deduced order of strand invasion (strand polarity). Confirmation by gel blot analysis is indicated for several sites. [file 1741-7007-9-64-S3.DOC]

| **Table S1.** Changes in coverage and putative strand invasion polarity for intermediate repeats in the *msh1* mutant. Coverage data are shown for first and advanced generation mutants. The data show changes in coverage with respect to Col-0 for regions flanking each repeat, with deduced order of strand invasion (strand polarity). Confirmation by gel blot analysis is indicated for several sites.  **Repeat *msh1* first generation* *msh1* advanced Strand polarity Blot data** |  |  |  |  |  |  |
| --- | --- | --- | --- | --- | --- | --- |
| A | 1 | 1 | 0.94 | 1.05 | 4 >1 | Yes |
|  | 0.77 | 1 | 0.94 | 1.1 |  |  |
| B | 0.88 | 0.85 | 1.09 | 1.05 | 1 >3 | Yes |
|  | 1 | 0.79 | 1.16 | 0.87 |  |  |
| C | 0.89 | 0.96 | 0.78 | 0.53 | 1 >4 | Yes |
|  | 0.93 | 0.98 | 0.95 | 1.11 |  |  |
| D | 0.73 | 0.64 | 0.86 | 0.77 | 3>2 | Yes |
|  | 1.08 | 0.85 | 0.85 | 0.5 |  |  |
| E | 0.58 | 0.39 | 0.29 | 0.21 | 4>1 | Yes |
|  | 0.69 | 0.89 | 0.09 | 0.6 |  |  |
| F | 1.32 | 0.98 | 2 | 1.17 | 4>1 | Yes |
|  | 0.83 | 1.22 | 0.12 | 1.17 |  |  |
| G | 0.42 | 0.72 | 0.07 | 0.55 | 2>4 | Yes |
|  | 0.69 | 1.23 | 0.89 | 1.42 |  |  |
| H | 0.56 | 0.46 | 0.82 | 0.93 | 4>2 | Yes |
|  | 0.7 | 0.83 | 0.03 | 0.41 |  |  |
| I | 0.82 | 0.43 | 0.95 | 0.21 | 1>4 | Yes |
|  | 0.68 | 1.03 | 0.32 | 0.94 |  |  |
| J | 1.03 | 0.97 | 1.11 | 0.51 | 4>1 | No |
|  | 0.46 | 0.58 | 0.27 | 0.83 |  |  |
| K | 0.78 | 0.91 | 0.62 | 0.77 | 2>4 | Yes |
|  | 0.79 | 0.72 | 0.81 | 0.68 |  |  |
| L | 0.35 | 0.62 | 0.12 | 0.77 | 2>3 | Yes |
|  | 0.72 | 0.61 | 0.65 | 0.06 |  |  |
| M | 1.07 | 1.09 | 1.87 | 1.32 | 4>1 | No |
|  | 0.36 | 0.34 | 0.09 | 0.04 |  |  |
| N | 0.81 | 0.96 | 0.43 | 0.76 | 2>3 | Yes |
|  | 0.93 | 0.76 | 1.44 | 1.23 |  |  |
| O | 0.9 | 0.83 | 0.69 | 0.14 | 2>4 | Yes |
|  | 0.81 | 0.8 | 0.69 | 0.85 |  |  |
| P | 1.17 | 1.18 | 1.18 | 1.21 | 4>1 or 1>4 | No |
|  | 0.3 | 0.31 | 0 | 0 |  |  |
| Q | 0.87 | 0.7 | 0.86 | 0.91 | 3>1 | Yes |
|  | 0.89 | 0.69 | 0.82 | 0.5 |  |  |
| R | 0.83 | 0.89 | 0.68 | 0.43 | 1>4 | Yes |
|  | 0.64 | 0.92 | 0.48 | 0.98 |  |  |
| S | 1.09 | 0.96 | 1.22 | 1.17 | 4>1 | No |
|  | 0.34 | 0.46 | 0 | 0 |  |  |
| T | 0.89 | 0.84 | 0.82 | 0.69 | 3>2 | yes |
|  | 0.8 | 0.68 | 0.93 | 0.66 |  |  |
| U | 0.77 | 0.92 | 0.65 | 0.68 | 2>4 | No |
|  | 0.98 | 0.94 | 1.17 | 1.07 |  |  |
| V | 1.11 | 1.12 | 1.1 | 1.52 | 4>1 | Yes |
|  | 0.77 | 0.66 | 0.71 | 0.53 |  |  |
| W | 0.77 | 0.69 | 0.71 | 0.08 | 1>3 | Yes |
|  | 1.21 | 1.05 | 1.33 | 0.72 |  |  |
| X | 0.86 | 0.89 | 0.81 | 0.91 | 2>3 | Yes |
|  | 0.96 | 1 | 0.88 | 0.92 |  |  |
| Z | 0.89 | 0.92 | 0.98 | 0.94 | 1>3 | Yes |
|  | 1.19 | 1.25 | 0.99 | 1.05 |  |  |
| AA | 0.69 | 0.47 | 0.44 | 0.07 | 1>4 | Yes |
|  | 1.14 | 1.13 | 1.07 | 1.38 |  |  |
| CC | 0.73 | 0.83 | 0.01 | 0.43 | 2>3 | No |
|  | 1.06 | 1.03 | 1.8 | 1.01 |  |  |
| DD | 1.02 | 0.84 | 0.98 | 0.81 | 1>4 | yes |
|  | 1.24 | 1.35 | 1.65 | 1.9 |  |  |
| EE | 1.03 | 0.97 | 0.9 | 1.03 | 2>4 | No |
|  | 0.98 | 0.95 | 0.94 | 0.9 |  |  |
| GG | 0.95 | 0.8 | 0.35 | 0.31 | 1>3 | Yes |
|  | 1 | 1.08 | 0.83 | 1.02 |  |  |
| HH | 0.95 | 0.89 | 0.44 | 0.67 | 2>3 or 4>1 | No |
|  | 1.02 | 0.97 | 0.65 | 0.99 |  |  |
| II | 0.94 | 0.94 | 0.93 | 1.1 | 4>1 | No |
|  | 0.92 | 1.09 | 0.84 | 1.05 |  |  |
| JJ | 0.95 | 0.82 | 0.62 | 0.48 | 1>3 | No |
|  | 1.18 | 1.28 | 1.31 | 1.38 |  |  |
| KK | 0.92 | 1 | 0.79 | 0.98 | 2>3 | No |
|  | 1.12 | 1 | 0.98 | 0.84 |  |  |
| MM | 0.99 | 0.92 | 0.29 | 0.38 | 3>2 | No |
|  | 0.57 | 0.59 | 1 | 0.22 |  |  |
| NN | 1.01 | 0.97 | 0.33 | 0.34 | 2>3 | No |
|  | 1 | 1.11 | 1.26 | 1.07 |  |  |
| OO | 1.12 | 1.09 | 0.42 | 0.49 | 2>3 | No |
|  | 0.9 | 0.72 | 1.03 | 0.78 |  |  |
| PP | 1.05 | 1.28 | 2.07 | 2.11 | 4>2 | No |
|  | 0.96 | 1.05 | 1.04 | 1.14 |  |  |
| QQ | 1.27 | 1.32 | 1.12 | 1.17 | 2>4 | No |
|  | 1.09 | 1.02 | 1.22 | 1.15 |  |  |
| RR | 1.16 | 1.22 | 0.66 | 0.73 | 3>1 | No |
|  | 0.84 | 0.85 | 0.89 | 0.02 |  |  |
| SS | 1.12 | 1.11 | 1.57 | 2 | 3>1 | Yes |
|  | 1.03 | 1 | 0.95 | 0.95 |  |  |
| TT | 1.08 | 1.15 | 1.09 | 1.19 | 3>1 | No |
|  | 1.01 | 1.09 | 1.03 | 0.9 |  |  |
| UU | 1.04 | 0.98 | 1.04 | 1.13 | 2>3 | No |
|  | 1.17 | 1.12 | 1.16 | 1.08 |  |  |
| VV | 0.65 | 0.8 | 0.73 | 0.82 | 4>1 | No |
|  | 1.02 | 1.07 | 0.89 | 0.87 |  |  |
|  |  |  |  |  |  |  |

*orientation shown is region1----region 2/ region3-----region 4. Some repeats are present in inverted orientation, accounting for 1>3, 3>1, 2>4 or 4>2 polarities.
