# Supplementary material for: Double-strand break repair processes drive evolution of the mitochondrial genome in Arabidopsis
Source: BMC Biol. 2011 Sep 27;9:64. doi: 10.1186/1741-7007-9-64 (PMC3193812; doi:10.1186/1741-7007-9-64)
Supplement: Additional file 5 — Table S2. Primers used in the study. [file 1741-7007-9-64-S5.DOC]

Table S2. Primers used in the study

| **AGI** | **Forward Primer (5’-3’)** | **Reverse Primer (3’-5’)** |
| --- | --- | --- |
| ATMG00620-orf139b | ATCGGCCAATCACGCTATCTCCTT | TTCCCTCGTGTAGCTATGATGCCA |
| ATMG00180-ccb452 | TGGTGCGGCTTAGGTCAACTAACA | TCGCAAACAACAACGTTCCTTCCC |
| ATMG00210-rpl5 | GATTCCGCGCGGTCAGAAATTCAT | AGTGCTTTGTCGTGCTAGGTCACT |
| ATMG00220-cob | AAGCTTAGCTAGCGCCATACCTGT | ATGCAATGCGGCCAGATGAAGAAG |
| ATMG00080-rpl16 | ACCGGGAAACCCACAGAAGTAAGA | TCGAGCATTTGCCAAACTCACACC |
| ATMG00480-atp8 | TACGGAACCAACTGCTTTCACACC | ACGGCCTTACACCATTGGGATACT |
| RL | GGGACTAGCCCGCTTCTTCATTAAA | ATAAGAAAGTGGAGGCAGGCTTGG |
| RQ | TGAGGAGAATGGGAGTTTGTGGGT | ATCTCAGGTTTACACTGCTAGATCA |
| RV | TGTTGGTCAACAACCACCCACAAC | TGATTCCCTCCAGACAGCTTCACT |
| 1.2 deletion-LJ | TCCCTCTCTACAGTGAGGTTCTGT | TTCCGGTCGCGCTCTTCTTCTAA |
| 1.2 deletion-RJ | GTCGTGAACATTTCTCCAACCCGT | AGGTAAGCCAGGTAAGCCATGTCA |
| 1.7 deletion | AAGACTCCGACAATAGGCGATTCC | AGTCGAGTTCTTCCCTCTCGTT |
| RealAct2 | TGTTGCCATTCAGGCCGTTCTTTC | ACAGTGTGAGACACACCATCACCA |
| RealMsh1 | TCATGCGTGTATGTG ATGCGGAGA | ACTTGACCCTTGCAGTCCTTCCTT |
